# High-Resolution Peripheral Quantitative Computed Tomography for Bone Evaluation in Inflammatory Rheumatic Disease

**DOI:** 10.3389/fmed.2020.00337

**Published:** 2020-07-15

**Authors:** Rasmus Klose-Jensen, Justin J. Tse, Kresten Krarup Keller, Cheryl Barnabe, Andrew J. Burghardt, Stephanie Finzel, Lai-Shan Tam, Ellen-Margrethe Hauge, Kathryn S. Stok, Sarah L. Manske

**Affiliations:** ^1^Department of Rheumatology, Aarhus University Hospital, Aarhus, Denmark; ^2^Department of Clinical Medicine, Faculty of Health, Aarhus University, Aarhus, Denmark; ^3^Cumming School of Medicine, McCaig Institute for Bone and Joint Health, University of Calgary, Calgary, AB, Canada; ^4^Department of Radiology, Cumming School of Medicine, University of Calgary, Calgary, AB, Canada; ^5^Diagnostic Centre, Silkeborg Regional Hospital, Silkeborg, Denmark; ^6^Department of Medicine, Cumming School of Medicine, University of Calgary, Calgary, AB, Canada; ^7^Department of Radiology and Biomedical Imaging, University of California, San Francisco, San Francisco, CA, United States; ^8^Department of Rheumatology and Clinical Immunology, Medical Centre - University of Freiburg, Freiburg, Germany; ^9^Faculty of Medicine, University of Freiburg, Freiburg, Germany; ^10^Department of Medicine and Therapeutics, The Chinese University of Hong Kong, Hong Kong, China; ^11^Department of Biomedical Engineering, The University of Melbourne, Parkville, VIC, Australia

**Keywords:** HR-pQCT (high-resolution peripheral quantitative computed tomography), arthritis, joint space, erosions, osteophytes, bone mineral density, bone microstructure

## Abstract

High resolution peripheral quantitative computed tomography (HR-pQCT) is a 3-dimensional imaging modality with superior sensitivity for bone changes and abnormalities. Recent advances have led to increased use of HR-pQCT in inflammatory arthritis to report quantitative volumetric measures of bone density, microstructure, local anabolic (e.g., osteophytes, enthesiophytes) and catabolic (e.g., erosions) bone changes and joint space width. These features may be useful for monitoring disease progression, response to therapy, and are responsive to differentiating between those with inflammatory arthritis conditions and healthy controls. We reviewed 69 publications utilizing HR-pQCT imaging of the metacarpophalangeal (MCP) and/or wrist joints to investigate arthritis conditions. Erosions are a marker of early inflammatory arthritis progression, and recent work has focused on improvement and application of techniques to sensitively identify erosions, as well as quantifying erosion volume changes longitudinally using manual, semi-automated and automated methods. As a research tool, HR-pQCT may be used to detect treatment effects through changes in erosion volume in as little as 3 months. Studies with 1-year follow-up have demonstrated progression or repair of erosions depending on the treatment strategy applied. HR-pQCT presents several advantages. Combined with advances in image processing and image registration, individual changes can be monitored with high sensitivity and reliability. Thus, a major strength of HR-pQCT is its applicability in instances where subtle changes are anticipated, such as early erosive progression in the presence of subclinical inflammation. HR-pQCT imaging results could ultimately impact decision making to uptake aggressive treatment strategies and prevent progression of joint damage. There are several potential areas where HR-pQCT evaluation of inflammatory arthritis still requires development. As a highly sensitive imaging technique, one of the major challenges has been motion artifacts; motion compensation algorithms should be implemented for HR-pQCT. New research developments will improve the current disadvantages including, wider availability of scanners, the field of view, as well as the versatility for measuring tissues other than only bone. The challenge remains to disseminate these analysis approaches for broader clinical use and in research.

## Introduction

**Figure 1:**
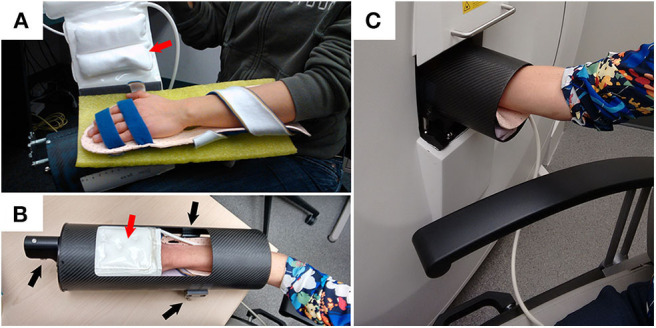
Example demonstrating how a participant is positioned for XtremeCTII scanning. **(A)** Participant's hand is positioned on a rigid-formed mold, with straps securing the distal and proximal ends of the forearm. An additional x-ray compatible, inflatable, pad-based positioning system (red arrows) is positioned above the hand to further reduce accidental motion during scan acquisitions. **(B)** The secured appendage is then placed within a cylindrical, annular, carbon-fiber molded sample holder. Using three spherical landmarks (black arrows), and with the participant resting comfortably, the supported participants' hand and sample holder is then securely and accurately placed within the scanner **(C)**.

Imaging is playing an increasing role in the diagnosis and monitoring of inflammatory disease. For many years the only imaging modality for joint assessment was conventional radiographs (CR); however, newer technologies have revolutionized the diversity of medical imaging capabilities in rheumatic diseases. Magnetic resonance imaging (MRI) and ultrasound (US) are now used regularly in both research and clinical practice for investigating joint diseases, particularly for the identification of soft tissue pathology and inflammation. In contrast, computed tomography (CT) capitalizes on the ability of bone to attenuate x-rays, thus providing excellent bone contrast in three dimensions with high spatial resolution but is limited by poor soft-tissue contrast. High-resolution peripheral quantitative computed tomography (HR-pQCT) imaging was designed to examine volumetric bone mineral density and microstructure of the radius and tibia and has been used extensively in osteoporosis research. HR-pQCT uses the same principles as traditional CT but can achieve a much higher spatial resolution and still has a very low radiation dose. The total effective dose for the metacarpophalangeal (MCP) joint and wrist scans is ~0.025 mSv (1). For comparison, the radiation dose for a conventional chest X-ray is 0.1 mSv. Still, HR-pQCT has a smaller field of view. Isotropic voxel sizes of 61 or 82 μm lead to spatial resolutions of 100 or 142 μm, respectively, approximately the thickness of an individual human trabecula. The high resolution allows segmentation of the bone at the microstructural level, permitting quantification of architecture and micro-scale pathological features, such as erosions. Initially limited by a gantry size that limited imaging to distal joints in the hands (Figure 1), wrist, foot, and ankles only, the newest generation HR-pQCT permits more proximal imaging of the elbows and knee, expanding capabilities to important anatomic sites in the context of inflammatory arthritis.

In 2011 the Study grouP for x-trEme Computed Tomography in Rheumatoid Arthritis (SPECTRA) was formed to facilitate the worldwide collaboration of periarticular image acquisition standards, image interpretation and analysis guides, and applications in clinical research. A systematic review was published in 2016, detailing the published research of HR-pQCT imaging and arthritis (2). Since then, the number of published papers has more than doubled, suggesting the importance of this review to update the current knowledge and expanded uses of this technology. We have structured this review to summarize pathological findings observed at the metacarpophalangeal (MCP) and wrist joints in different types of peripheral inflammatory arthritis using HR-pQCT, compare HR-pQCT imaging outcomes with other modalities in terms of erosions, joint space width, bony proliferations, bone density and microstructure, summarize the reproducibility of the quantitative outcome measures assessed with HR-pQCT imaging, investigate early detection of arthritis, the longitudinal changes over time for the quantitative outcome measures, and finally discuss the implications of the use of HR-pQCT in inflammatory arthritis and future directions.

## Materials and Methods

### Search Strategy

US National Library of Medicine Medical Subject Heading (MeSH) terms, keywords, and acronyms for HR-pQCT were selected. We combined this search with MeSH terms, keywords, and acronyms for the metacarpophalangeal joints (MCP) and/or radius and/or wrist, to specify the periarticular regions of interest, and MeSH terms and keywords for arthritis (search terms available in Appendix 1 in Supplementary Material). PubMed (1966–January 2020) and Embase (1980–January 2020) searches were conducted to identify potentially relevant studies. Filters were applied to eliminate animal studies and identify English language studies and full-length articles. Web of Science was used to identify articles which referenced the articles found through PubMed and Embase. References from identified articles were checked manually. Authors from the SPECTRA Collaboration aided in identifying any potential studies we did not find through the other searches.

### Inclusion Criteria

Any studies reporting original results of HR-pQCT imaging of the MCP and/or wrist joints were selected through the title/abstract search. These studies could report on the normal state or any arthritis conditions [including RA, osteoarthritis (OA), PsA], as well as “pre-arthritis” states [e.g., persons positive for anticitrullinated protein antibodies (ACPA) with arthralgia]. At the full-text review stage, we selected articles for data extraction if they reported on any of the following outcomes: (1) pathology findings, determined a priori to include bone mineral architecture, bone mineral density (BMD), erosions, vascular channels, cortical breaks, joint space, bony proliferations, or surface changes; (2) comparison to other imaging modalities; and (3) reproducibility. Article selection and data extraction were performed by one author (RKJ).

### Analysis

Owing to the heterogeneity of case definitions for different pathologies, variations in analysis techniques, and the identification of studies in a variety of normal and disease states, it was not possible to perform a meta-analysis. Therefore, a narrative summary of the work was performed following pathology descriptions and comparisons to other imaging modalities and reproducibility.

## Pathology

**Figure 2:**
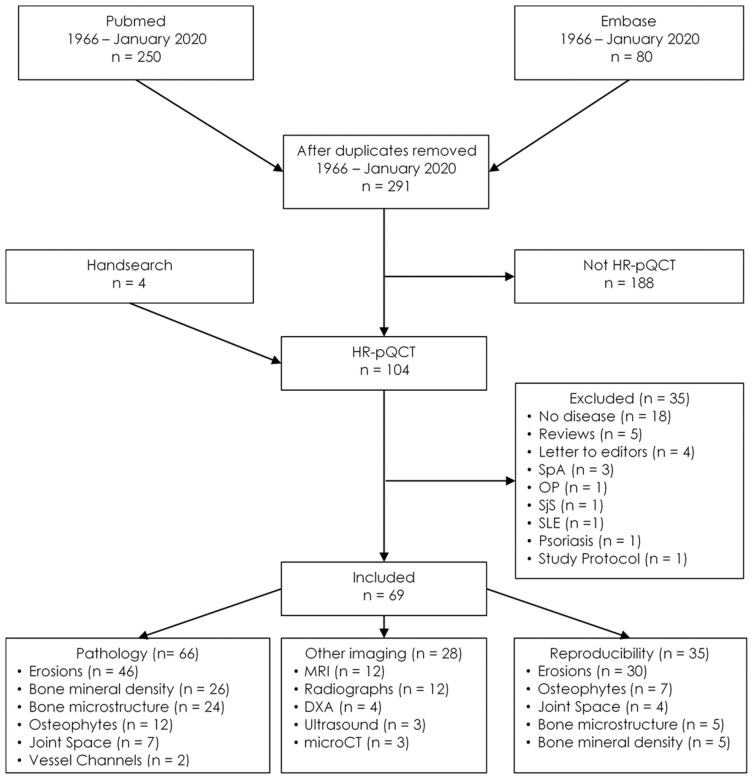
Flow diagram for study inclusion. DXA, Dual x-ray absorptiometry; HR-pQCT, High-resolution peripheral quantitative computed tomography; OP, Osteoporosis; SjS, Sjogren's syndrome; SpA, Spondyloarthritis; SLE, Systemic lupus erythematosus; microCT, Micro-computed tomography.

A total of 291 unique publications were identified, each subject to selection based on the title and abstract. One hundred and three met eligibility for full-text review, with 69 selected for data extraction (Figure 2). Of these 69 articles, 66 described pathology findings, 28 related comparisons of HR-pQCT findings to another imaging modality, and 35 described precision or reproducibility. All the studies meeting inclusion criteria are detailed in Table 1.

**Table 1:** Studies included in the systematic review.

**Author, year (ref)**	**Participants**	**Type of study**	**Joints included**	**Outcomes**	**Other modalities**
Fouque-Aubert, 2010 (3)	RA (*n* = 57); eRA (*n* = 36); control (*n* = 43)	Cross-sectional	MCP	Erosions, BMD, microstructure	
Stach, 2010 (4)	RA (*n* = 58); control (*n* = 30)	Cross-sectional	MCP; Radius	Erosions, Osteophyte	
Finzel, 2011 (5)	RA (*n* = 58); PsA (*n* = 30)	Cross-sectional	MCP	Erosions, Osteophyte	
Finzel, 2011 (6)	RA (*n* = 30)	Cohort	MCP	Erosions	
Finzel, 2011 (7)	RA (*n* = 14); PsA (*n* = 6); control (*n* = 6)	Cross-sectional	MCP	Erosions	US
Zhu, 2012 (8)	RA (*n* = 100)	Cross-sectional	MCP; Radius	BMD, microstructure	DXA
Albrech, 2013 (9)	RA (*n* = 50)	Cross-sectional	MCP	Erosions	MRI
Aschenberg, 2013 (10)	RA (*n* = 40)	Cohort	MCP	Erosions, Osteophyte	
Barnabe, 2013 (11)	eRA (*n* = 10)	Cross-sectional	MCP	JSW	
Barnabe, 2013 (12)	RA (*n* = 15); control (*n* = 15)	Cross-sectional	MCP; PIP	Erosions, JSW, BMD, microstructure	CR
Burghardt, 2013 (13)	RA (*n* = 16); control (*n* = 7)	Cross-sectional	MCP; Radius	JSW	CR
Finzel, 2013 (14)	PsA [(*n* = 41) TNFi/MTX *n* = 28/13]	Cohort	MCP	Erosions, Osteophyte	
Finzel, 2013 (15)	RA (*n* = 20)	Cohort	MCP	Erosions	
Srikhum, 2013 (16)	RA (*n* = 16); control (*n* = 7)	Cross-sectional	MCP; Radius	Erosions	MRI
Zhu, 2013 (17)	RA (*n* = 66); control (*n* = 66)	Cross-sectional	Radius	BMD, microstructure	DXA
Kleyer, 2014 (18)	ACPA+ (*n* = 15); ACPA– (*n* = 15)	Cross-sectional	MCP	BMD, microstructure	
Kocijan, 2014 (19)	RA (*n* = 90); control (*n* = 70)	Cross-sectional	Radius	BMD, microstructure	
Kocijan, 2014 (20)	RA (*n* = 60); PsA (*n* = 50)	Cross-sectional	Radius	BMD, microstructure	
Finzel, 2014 (21)	PsA (*n* = 25); HOA (*n* = 25); control (*n* = 20)	Cross-sectional	MCP	Osteophyte	
Teruel, 2014 (22)	RA (*n* = 16)	Cross-sectional	Radius	BMD, microstructure	MRI
Töpfer, 2014 (23)	RA (*n* = 18)	Cross-sectional	MCP	Erosions	
Zhu, 2014 (24)	RA (*n* = 50); control (*n* = 50)	Cross-sectional	Radius	BMD, microstructure	
Zhu, 2015 (25)	PsA (*n* = 53); control (*n* = 53)	Cross-sectional	Radius	BMD, microstructure	DXA
Hecht, 2015 (26)	RA (*n* = 242)	Cross-sectional	MCP	Erosions	
Kocijan, 2015 (27)	PsA (*n* = 50); PsO (*n* = 30); control (*n* = 70)	Cross-sectional	Radius	BMD, microstructure	
Lee, 2015 (1)	RA (*n* = 16)	Cross-sectional	MCP; Radius	Erosions	MRI; CR
Regensburger, 2015 (28)	RA (*n* = 103)	Cross-sectional	MCP; Radius	Erosions	MRI
Töpfer, 2015 (29)	RA (*n* = 22)	Cohort	MCP	Erosions, BMD	
Barnabe, 2016 (30)		Cross-sectional	MCP	Erosions	
Figueriredo, 2016 (31)	ACPA+ RA (*n* = 202)	Cohort	MCP	Erosions, Osteophytes	
Kleyer, 2016 (32)	ACPA+ (*n* = 20); ACPA– (*n* = 13)	Cross-sectional	MCP	Erosions	MRI
Scharmga, 2016 (33)	RA (*n* = 34); eRA (*n* = 10); control (*n* = 38)	Cross-sectional	MCP	Erosions	MRI; CR
Scharmga, 2016 (34)	Cadaver (*n* = 10)	Cross-sectional	MCP; PIP	Erosions	μCT
Shen, 2016 (35)	PsA (*n* = 80)	Cross-sectional	Radius	BMD, BMS	DXA
Tom, 2016 (36)	Cadaver (*n* = 7)	Cross-sectional	MCP	JSW	
Feehan, 2017 (37)	eRA (*n* = 30); control (*n* = 30)	Cohort	MCP; Radius	BMD, BMS	
Kleyer, 2017 (38)	RA (*n* = 15); PsA (*n* = 15); control (*n* = 15)	Cross-sectional	MCP	3D printing	
Peters, 2017 (39)	RA (*n* = 7); control (*n* = 3)	Cross-sectional	MCP	Erosions	
Peters, 2017 (40)	RA (*n* = 32); control (*n* = 32)	Cohort	MCP	Erosions	μCT
Scharmga, 2017 (41)	Cadaver (*n* = 7)	Cross-sectional	MCP	Erosions, VC	
Shimizu, 2017 (42)	RA [(*n* = 27) TNFi/MTX *n* = 17/10]	Cohort	MCP; Radius	Erosions, JSW, BMD	MRI; CR
Simon, 2017 (43)	RA (*n* = 106); control (*n* = 108); Cadaver (*n* = 6)	Cross-sectional	MCP; Radius	BMD, BMS	
Werner, 2017 (44)	RA (*n* = 107); control (*n* = 105); Cadaver (*n* = 6)	Cross-sectional	MCP; Radius	Erosions, VC	μCT
Yang, 2017 (45)	RA (*n* = 12); control (*n* = 20)	Cross-sectional	MCP; Radius	BMD, microstructure	
Yue, 2017 (46)	RA (*n* = 20); RA (*n* = 20)	Cohort	MCP	Erosions	
Figueriredo, 2018 (47)	RA (*n* = 65)	Cross-sectional	MCP	Erosions	
Ibrahim-Nasser, 2018 (48)	RA (*n* = 29)	Cross-sectional	MCP	Erosions, BMD, microstructure	CR
Kampylafka, 2018 (49)	PsA [(*n* = 20) IL17 = 20)	Cohort	MCP; PIP; Radius	Erosions, Osteophytes, BMD, microstructure	MRI; US
Keller, 2017 (50)	Control [ACPA+ (*n* = 29); ACPA– (*n* = 29)]	Cross-sectional	MCP	Erosions, BMD, microstructure	
Kong, 2018 (51)	RA [(*n* = 32) US+/– *n* = 20/12]	Cross-sectional	MCP	BMD, microstructure	US
Peters, 2018 (52)	RA (*n* = 41); control (*n* = 38)	Cross-sectional	MCP; PIP	Erosions	MRI; CR
Peters, 2018 (53)	eRA (*n* = 17); undifferentiated arthritis (*n* = 4)	Cross-sectional	MCP	Erosions	
Scharmga, 2018 (54)	RA (*n* = 39); control (*n* = 38)	Cross-sectional	MCP; PIP	Erosions	MRI; CR
Scharmga, 2018 (55)	RA (*n* = 20); control (*n* = 10)	Cross-sectional	MCP; PIP	Erosions	CR
Simon, 2018 (56)	RA ACPA + (*n* = 106); RA ACPA – (*n* = 30); CD (*n* = 43); UC (*n* = 27); PsO (*n* = 74); PsA (*n* = 88); control (*n* = 108)	Cross-sectional	MCP	BMD, microstructure	
Simon, 2018 (57)	PsA (*n* = 55); PsO (*n* = 55); control (*n* = 47)	Cross-sectional	MCP	Erosions, Osteophytes	
Yue, 2018 (58)	eRA (*n* = 63); Remission/not remission	Cohort	MCP	Erosions, BMD, microstructure	
Finzel, 2019 (59)	eRA (*n* = 66 TOC/TNFi *n* = 33/33)	Cohort	MCP; Radius	Erosions	
Berlin, 2019 (60)	Control (*n* = 120)	Cross-sectional	MCP; PIP	Erosions, Osteophytes	
Henchie, 2019 (61)	RA (*n* = 17); PsA (*n* = 17); control (*n* = 12)	Cross-sectional	MCP	Erosions, Osteophytes	
Keller, 2019 (62)	Control [ACPA+ (*n* = 22); ACPA– (*n* = 23)]	Cohort	MCP; Radius	Erosions	
Manske, 2019 (63)	RA (*n* = 43)	Cross-sectional	MCP	JSW	CR
Peters, 2019 (64)	RA (*n* = 32); control (*n* = 32)	Cohort	MCP; PIP	Erosions, BMD, microstructure	CR
Shimizu, 2019 (65)	RA [(*n* = 28) TNFi+/– *n* = 18/10]	Cohort	Radius	Erosions	MRI
Wu, 2019 (66)	PsA (*n* = 60)	Cohort	MCP	Erosions, Osteophytes.	
Yue, 2019 (67)	eRA (*n* = 117)	Cohort	MCP	Erosions	
Simon, 2019 (68)	PsA [(*n* = 165) None/MTX/bDMARD *n* = 79;52;34]	Cross-sectional	Radius	BMD, microstructure	
Wu, 2020 (69)	PsA (*n* = 62); control (*n* = 62)	Cross-sectional	MCP	Erosions, Osteophytes, BMD, microstructure	
Stok, 2020 (70)	RA (*n* = 30)	Cross-Sectional	MCP	JSW	

*RA, Rheumatoid arthritis; PsA, Psoriasis arthritis; PSO, Psoriasis; ACPA, Anticitrullinated protein antibodies; US, Ultrasound; IL, interleukin; TNFi, Tumor necrosis factor inhibitors; TOC, tocilizumab; UC, Ulcerative colitis; CD, Crohn's disease; MTX, Methotrexate; bDMARD, biological Disease-modifying antirheumatic drug; BMD, Bone mineral density; MCP, Metacarpophalangeal; PIP, proximal interphalangeal*.

### Erosion Detection by HR-pQCT

Bone erosions were originally defined radiographically as breaks in the cortical bone surface, typically accompanied by loss of underlying trabecular bone (71–73).

**Figure 3:**
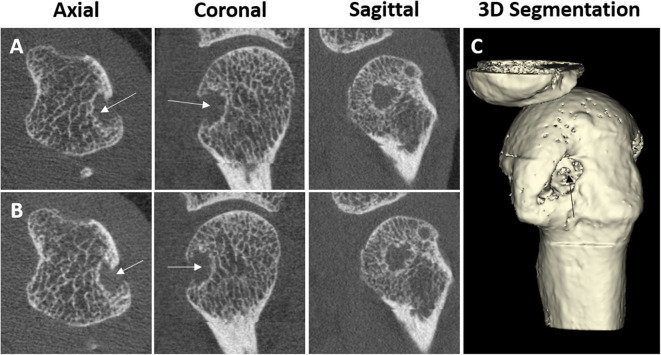
Example of a cortical break meeting the SPECTRA definition of erosion by high-resolution peripheral quantitative computed tomography imaging in the radial quadrant of the second metacarpophalangeal joint from a 54-year-old female patient with rheumatoid arthritis. Arrows indicate the cortical break. **(A,B)** Are the grayscale, consecutive slices. **(C)** Is the 3D segmentation, performed using thresholding (3D Slicer, http://www.slicer.org). Images provided courtesy of Aarhus University (RKJ, KKK, EMH).

Erosions on HR-pQCT have been defined in a diverse manner; the early studies simply defined erosion as a clear juxta-articular break of the cortical shell (4, 5) while others stated that the break had to be seen in a minimum number of consecutive slices (3). Not until 2016 was a clear definition proposed by the SPECTRA Collaboration following a consensus exercise (63). Erosions were defined as (1) a definite break in the cortical bone; (2) the cortical break must extend over at least two consecutive slices; (3) the cortical break must be detectable in two perpendicular planes; (4) the cortical interruption must have a loss of underlying trabecular bone, and (5) the cortical interruption must be non-linear in shape to differentiate from vascular channels penetrating the cortices (Figure 3). In contrast, cortical breaks or interruptions have been defined as a clear interruption of the cortex, seen on two consecutive slices on two orthogonal planes but without the need to demonstrate loss of trabecular bone or shape (34). Such, cortical breaks or interruptions have not been incorporated in the SPECTRA definition of erosions.

The high spatial resolution of HR-pQCT images has allowed deeper investigation into the role of these erosions in the inflammatory arthritis disease course. Forty-six publications characterizing erosions or cortical breaks/interruptions were found. Erosions have been assessed in a variety of arthritic conditions including early inflammatory arthritis (3, 33, 37, 53, 58, 59, 67), RA (1, 3–7, 9, 10, 12, 15, 23, 26, 28–30, 34, 39, 40, 44, 46–48, 52, 54, 55, 61, 64, 65, 67), PsA (5, 7, 14, 49, 61, 66, 69), and erosive hand OA (21), as well as healthy controls (3, 5, 7, 16, 33, 39, 40, 44, 45, 52, 54, 55, 60, 61, 64, 67) and subjects with ACPA antibodies but no features of arthritis (32, 50, 62).

Erosions are most abundant in patients with RA (3, 4, 16, 33, 52, 54, 55, 57, 64, 65, 69), especially ACPA and rheumatoid factor (RF) positive RA patients (26). The erosions exhibit a clear predilection for the radial and ulnar quadrant of the 2nd and 3rd MCP joints. Erosions are most common in the metacarpal head but are also prevalent in the proximal interphalangeal (PIP) bases (4, 15). Although patients with PsA also exhibited a high degree of erosive damage (69), erosions are not as prevalent in PsA patients when compared with RA patients (5, 7, 61). For patients with PsA, erosions are most common in the radial quadrant. However, PsA erosions were also found to a high degree in the dorsal and palmar quadrant of the MCP joint (5, 69). Differences in erosion morphology have also been described in RA compared with PsA patients. In one study, erosions in RA patients were typically U-shaped in appearance, while erosions in PsA patients are more often tubule and Ω-shaped (5); these findings have not been confirmed in other studies. The possible differences in shape may reflect different pathophysiological mechanisms, leading to greater erosion repair in PsA than RA, or alternatively, these morphological phenotypes may reflect differences in disease severity. Further investigation is needed to explore differences between morphologies observed in RA and PsA.

**Figure 4:**
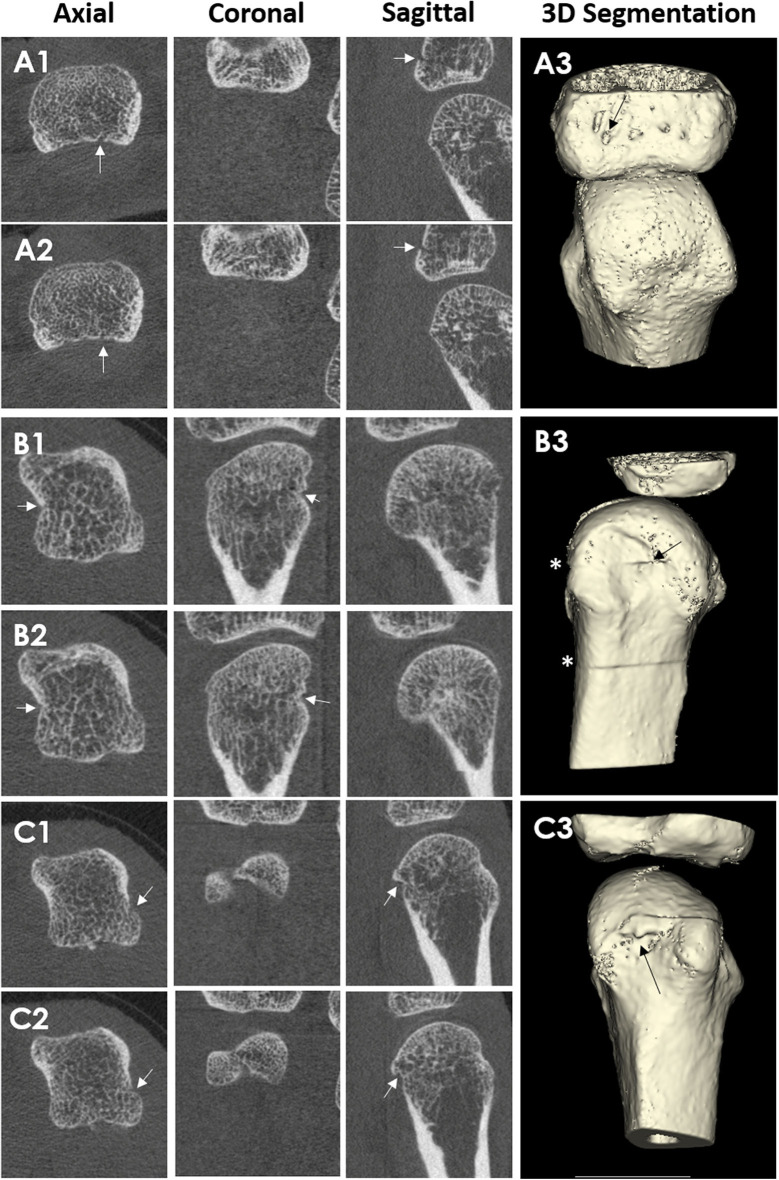
HR-pQCT images of cortical breaks, which do not meet the SPECTRA definition for erosions. Arrows indicate the cortical break. Patient A, where **(A1,A2)** are consecutive slices and **(A3)** is the 3D segmentation. Third MCP Joint from a 67-year-old male patient with rheumatoid arthritis. The cortical break is linear in shape. Therefore, the cortical break is defined as a vascular channel Patient B, where **(B1,B2)** are consecutive slices, and **(B3)** is the 3D segmentation. Second MCP Joint from a 51-year-old female patient with rheumatoid arthritis. Cortical break in relation to stack misalignment represented by *. Patient C, where **(C1,C2)** are consecutive slices and **(C3)** are 3D segmentation. Second MCP Joint from a 51-year-old female patient with rheumatoid arthritis. Cortical break in relation to a bony proliferation. Images provided courtesy of Aarhus University (RK-J, KK, and E-MH).

Typically, erosion presence has been assessed manually by an experienced reader. However, manual scoring is laborious and requires substantial training and standardization. An automated detection algorithm has been developed, named the Cortical Interruption Detection Algorithm (39, 40, 52, 53, 64). The Cortical Interruption Detection Algorithm detects the presence of cortical interruptions, directing the reader to lesion locations to verify that they meet the erosion definition as opposed to those that merely represent physiological breaks in the cortical bone, i.e., vascular channels (Figure 4A) or stack misalignment (Figure 4B) and osteophyte formation (Figure 4C). This algorithm also captures very small cortical breaks (>0.246 mm in diameter) that can be challenging to detect through visual inspection (39, 40).

### Comparison of Erosion Detection With Other Imaging Modalities

Erosion detection by HR-pQCT has been compared with other pre-clinical and clinical imaging modalities. An image review showing erosions on HR-pQCT, MRI, and CR have also been published for patients with RA (33).

Micro-computed tomography (microCT) applies the same technology as HR-pQCT for pre-clinical investigations but uses a smaller field of view to acquire even higher spatial resolution images (<10 μm, depending on the size of the sample and scanner characteristics) that can be performed on *ex vivo* samples only. Two studies compared cortical interruption detection using HR-pQCT with microCT as a gold standard. The sensitivity of HR-pQCT in detecting cortical interruptions was 82% (34). MicroCT has also been used as a gold standard in order to investigate the ability of the Cortical Interruption Detection Algorithm to find the minimum diameter to detect and quantify cortical interruptions on HR-pQCT. This algorithm performed best for the detection of cortical interruptions with a minimum diameter of 0.16 mm (40).

**Figure 5:**
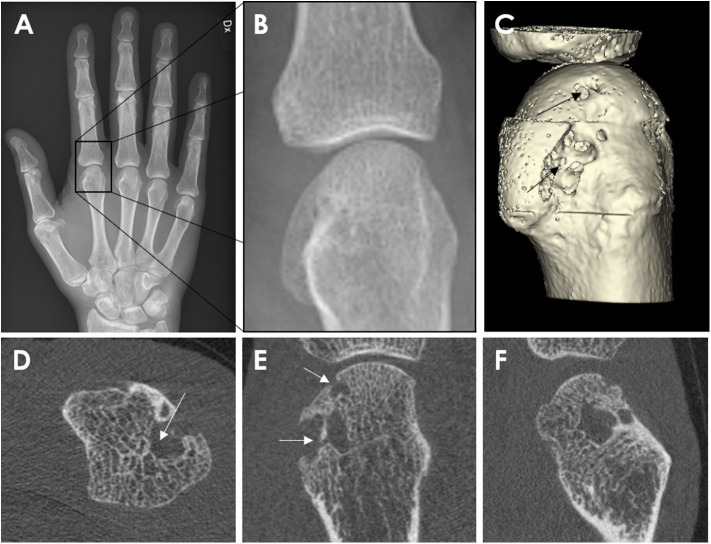
Conventional Radiography **(A,B)** and HR-pQCT imaging **(C–E)** from a 62-year-old male patient with rheumatoid arthritis. Anterior-posterior view of the right hand **(A)**. Enlargement of the second metacarpophalangeal joint **(B)**. High-resolution peripheral quantitative computed tomography images of the second MCP joint as 3D segmentations performed using thresholding **(C)** (3D Slicer, http://www.slicer.org) and the axial **(D)**, coronal **(E)**, and sagittal **(F)** plane, congruent with the corresponding radiograph. The arrow demonstrates the erosion location. Images provided courtesy of Aarhus University (RKJ, KKK, EMH).

Additional research has been performed comparing clinical CR and MRI with HR-pQCT, as a gold standard, for the detection of erosions (Figure 5). In a clinical setting, CR is still the gold standard. The sensitivity of erosion detection by CR was 61 to 68% when HR-pQCT was considered as a reference (1, 12). Face validity of HR-pQCT identification of erosions was supported by one study that reported that the Sharp/van der Heijde (SvH) score based on MCP and PIP joints using CR were significantly associated with the number and size of erosions by HR-pQCT (52). However, the HR-pQCT scanner has a much smaller field of view, and it remains unknown if the added benefit of the greater sensitivity can make up for the fewer joints examined.

Twelve studies have compared erosion detection by HR-pQCT and MRI, with HR-pQCT as the gold standard, in patients with RA, PsA as well as ACPA positive arthralgia patients without arthritis. The sensitivity for MRI erosion detection ranged from 60 to 86% (1, 9, 16, 28), while the specificity was 97 to 100% (1, 9, 28, 32). HR-pQCT was superior at detecting erosions of less than 10 mm^3^ (9).

HR-pQCT has also been used as a reference for erosion detection in order to compare it with US (7). US and HR-pQCT were consistent in the measurement of the width of erosions. However, there was a 10% false-negative rate, and 29% false-positive rate for erosion detection by US. False-negative results recorded with US were due to its limited resolution to detect cortical lesions <2 mm in width. It was interpreted that the false-positive results occurred because of surface irregularities suggestive of bone erosions on US (7). US has a crucial drawback, as some quadrants of the joints are hard to visualize due to the acoustic window, e.g., radial or ulnar quadrant of the 3rd MCP.

Overall, these findings indicate that HR-pQCT detects more erosions and cortical interruptions than any other clinical imaging modality. However, with increased spatial resolution, more cortical interruptions are observed; this likely includes more physiological cortical interruptions, i.e., vascular channels. However, the higher resolution could also help distinguish pathological from physiological cortical interruptions.

### Quantitative Evaluation of Erosions From HR-pQCT Imaging

**Figure 6:**
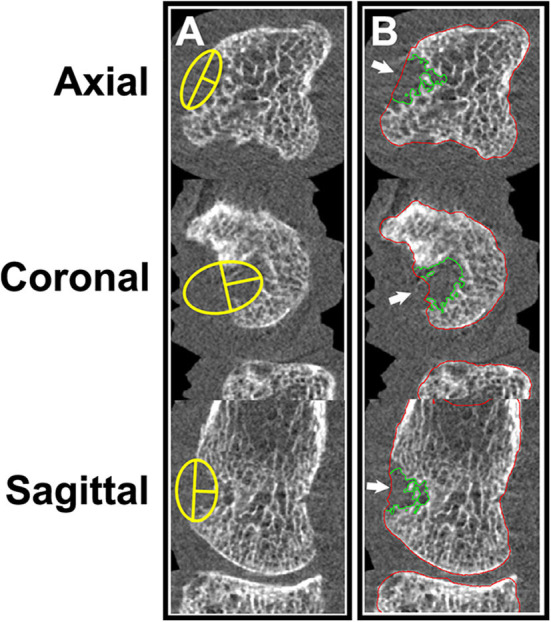
**(A)** Manual measurement of erosion width and depth. **(B)** Semi-automated erosion volume assessment using MIAF (University of Erlangen). Images provided courtesy of the University of Calgary (JJT, CB, SLM).

**Figure 7:**
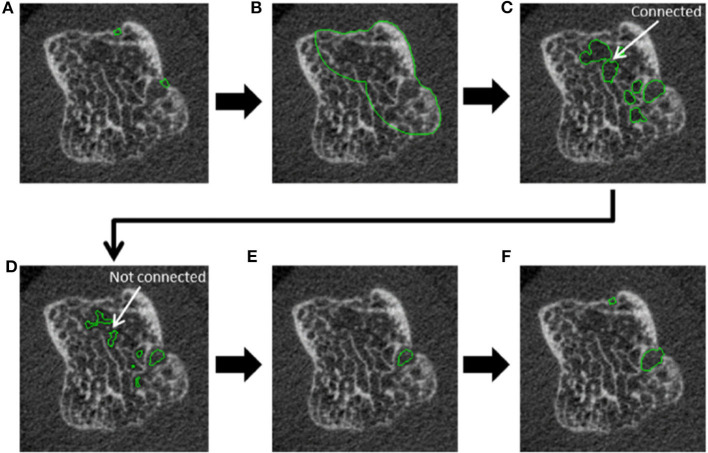
Steps incorporated in the automated cortical interruption detection algorithm and measurement of underlying loss of trabecular bone, as visualized on a 2D grayscale image. **(A)** Detection of two cortical interruptions ≥0.41 mm. **(B)** Region of interest (ROI) identified by dilating the cortical interruptions by 48 voxels (corresponding to 3.936 mm), and masking with the periosteal contour. **(C)** Only voids that are ≥0.738 in diameter are selected by performing a distance transformation within the ROI. **(D)** Voids are eroded by 2 voxels to detach connections of ≤ 0.328 mm and prevent leakage into the trabecular bone. **(E)** Inclusion of voids that remain connected to a cortical interruption after erosion. **(F)** Dilation of voids to the original size, and inclusion of cortical interruptions that were originally detected. Reproduced with permission from (53).

**Figure 8:**
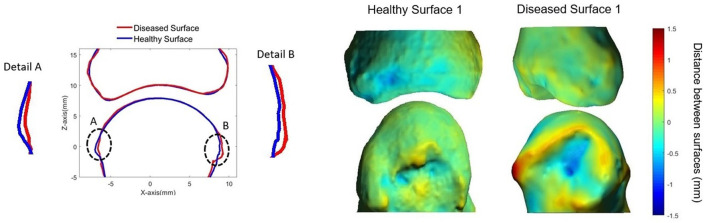
Surface deformation model (61) showing: **(Left)** cross-section of MCP joint with the diseased surface (red) overlaid on the average healthy surface (blue). **(Right)** 3D heat map representing distances between diseased (top) and healthy (bottom) surfaces. Positive distances reflect bony proliferations, while negative distances represent erosive damage. Image provided courtesy of Worcester Polytechnic Institute (Kyle Murdock and Karen Troy).

As a result of the high spatial resolution and 3D imaging, accurate quantification of erosion size is possible. Several published methods exist, including semiquantitative scores (1, 4, 16), manual measures of the maximal dimension of width and depth (5, 7, 15, 30, 46, 48, 58), sphericity (23, 29), and surface area (23, 52, 64). Erosion volume is the most commonly used method due to the ability to detect changes (progression or repair) in three dimensions. However, there is presently no consensus on how to quantify erosion volume. Erosion width and depth have been manually assessed using OsiriX medical imaging software (Pixmeo, Bernex, Switzerland) (48, 50, 62, 65). The erosion volume can then be estimated from a half-ellipsoid formula (9, 23, 26, 28, 47, 57) or ellipsoid formula (Figure 6A) (42). More recently, semi-automated and automated algorithms have been introduced. These include Medical Image Analysis Framework (MIAF) (custom software developed at the University of Erlangen; Figure 6B) (23, 29), modified Evaluation Script for Erosions (mESE; using Image Processing Language, Scanco Medical) (47), Cortical Interruption Detection Algorithm (custom analysis developed at Maastricht University and Eindhoven University of Technology using Image Processing Language, Scanco Medical; Figure 7) (39, 40, 52, 53, 64) and a surface transformation algorithm (custom software developed at Worcester Polytechnic Institute; Figure 8) (61). MIAF, mESE, and the Cortical Interruption Detection Algorithm perform a segmentation of the bone and an estimation of the location of the cortical break. Based on this segmentation, the number of voxels in the erosion are counted, producing a true volumetric measure of erosion size. In contrast, the surface transformation algorithm uses statistical shape modeling to compare surface deformities of inflammatory arthritis joints with healthy joints, producing measurements of erosion depth (Figure 8) (61).

A single study compared erosion volumes measured manually (estimated from width and depth and calculated assuming the erosion approximated a half-ellipsoid) and using MIAF and mESE. While erosion volumes were similar when measured with the semi-automated methods, manual measurements yield significantly lower absolute volume measures compared with semi-automated methods, particularly for irregularly shaped erosions, illustrating that the erosions should not be assumed to be shaped like a half-ellipsoid (47). To date, no other studies have compared other erosion measurement algorithms.

### Vascular Channels and Cortical Micro-Channels

The high resolution of HR-pQCT not only reveals cortical interruptions meet the definition of erosions but also interruptions which have been hypothesized to be vascular channels. According to the SPECTRA erosion definition, the cortical interruption was suspected to be vascular channels if the interruption was characterized by a parallel cortical lining seen on two consecutive slices and two orthogonal planes (41).

Efforts have been made to test this hypothesis. One study investigated 10 cadaveric fingers, not necessarily affected by inflammatory arthritis, imaged by HR-pQCT with subsequent histological evaluation. Fifty-two vascular channels were identified by histology, and 11 of these fulfilled the definition of erosions on HR-pQCT. Seven cortical breaks which fulfilled the SPECTRA definition of vascular channels were located. Five could be evaluated with histology, and only one of these was a true vascular channel. While they are not vascular channels, these microchannels may provide a link between the synovium and the bone marrow (41). Another approach for investigating vascular channels was to inject the contrast agent Imeron into the ulnar and radial artery of six cadaveric hands prior to HR-pQCT scanning (44). This technique detected very small intraarticular vascular channels. The vascular channels, however, were located proximal to the cortical micro-channels. With both studies, it is possible that very small vascular channels escaped detection due to the challenges of visualizing by perfusion and casting, microCT contrast or standard histological stains (44).

These findings do support the suggestion that bone has a large number of channels passing from the inside to the outside of the bone that is not necessarily linked to erosions, nor do all channels contain vessels. The shape of cortical interruptions cannot be used to accurately decide if cortical breaks contain vessels, as many cortical interruptions, that do not have a classic linear shape, contain vessels (41). Understanding the role of these various channels may be important to understand RA pathology. Traditionally, it has been postulated that synovitis is an initial process that is succeeded by bone involvement known as the “outside-in hypothesis.” Conversely, it has been suggested that RA is primarily a bone marrow disease which subsequently affects the synovial membrane; this is known as the “inside-out hypothesis” (74). The cortical micro-channels connect the synovial compartment with the bone marrow and might be important in investigating whether bone damage begins from the inside-out or outside-in (75, 76). HR-pQCT will be useful in combination with other imaging techniques to understand some of these pathological mechanisms.

### Bony Proliferations

**Figure 9:**
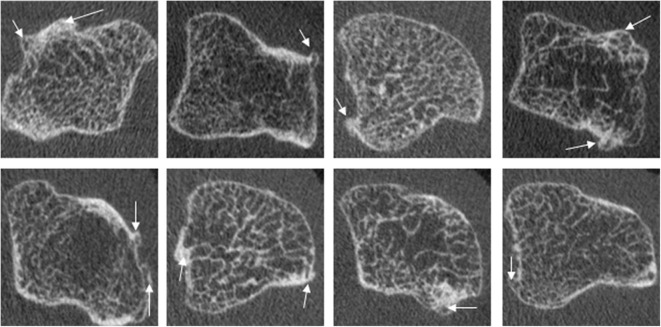
Examples of osteophytes in the metacarpal head of PsA patients, which commonly show widespread involvement of the cortical bone. Images provided courtesy of The Chinese University of Hong Kong (L-ST).

Bony proliferation is another common pathologic feature of many arthritic diseases, including PsA, primary hand OA and secondary OA due to inflammatory arthritis. There is currently no consensus on how to define or quantify the assessment of bony proliferations. However, most studies have defined osteophytes and enthesophytes in a similar fashion; these include bony protrusions from the juxta-articular cortical shell (4, 5, 10, 14, 21, 57) (Figure 9), bony proliferation at specific anatomical sites (57), or bone formation arising from the periosteal bone cortex at the insertion sites of the capsule, ligament, or tendons (66, 69). Thirteen publications characterizing osteophytes or enthesophytes with HR-pQCT were found; 8 of which were cross-sectional studies. In three of the studies, the osteophytes or enthesophytes were graded on a semi-quantitative scale (0–3) according to the height measured as the maximum distance between the original and the new cortical lining (4, 5, 21). Two of these three studies also presented direct measurements of the height. The remaining five studies measured osteophyte or enthesophyte height directly (5, 14, 21, 57), and one of these also segmented the osteophyte volume (69). Two studies found that chronic RA patients had a greater number and larger osteophytes than healthy subjects (4, 61); these osteophytes were found mostly in the palmar and dorsal quadrant of the joints. PsA patients had a greater number and size of osteophytes relative to RA patients (5, 61). The highest number of osteophytes in PsA patients was observed in the radial quadrant, closely followed by the dorsal and palmar quadrants. Enthesophytes in patients with PsA is more abundant and greater in size compared to healthy subjects. The majority of enthesophytes in patients with PsA were found at the palmar and dorsal quadrants of the metacarpal heads (57). The volume of enthesophytes is also significantly greater in patients with PsA compared to healthy controls (69). Patients with PsA had a similar number and size of osteophytes compared to those with hand OA. The osteophytes of patients with hand OA were seen predominantly in the palmar and dorsal quadrant, while the distribution of osteophytes in patients with PsA was more widespread (21). In addition, patients with PsA have significantly taller and more abundant bony proliferations (5). There have also been attempts to study bony proliferations according to age. In healthy subjects, the number of osteophytes in the 2nd and 3rd MCP and PIP joints were found to increase with age (60). The number and height of enthesophytes also increase with age; these have been observed not only in patients with PsA but also in healthy subjects. Therefore, the progression of osteophytes may not solely signify disease severity but may also indicate normal age-related change (57). Like erosions, the sensitivity for enthesophyte detection by HR-pQCT is far higher than MRI as 89% of PsA patients had enthesophytes by HR-pQCT, while they were observed in only 30% of the PsA patients on MRI (49). Presently, it is unknown whether there is a benefit of HR-pQCT imaging for tracking the progression of bony proliferation compared to conventional radiographs since no studies have compared the sensitivity and osteophytes seem to progress despite therapy.

### Joint Space Width

**Figure 10:**
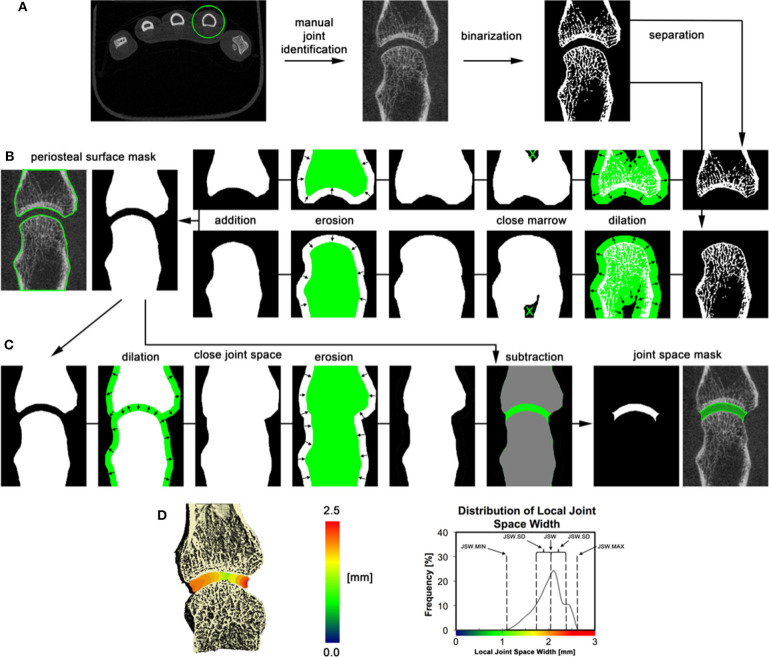
3D volumetric joint space width analysis by HR-pQCT. **(A)** The relevant joint is identified; bone is segmented by thresholding, and bones are separated based on their relevant position (distal and proximal). **(B)** Periosteal surfaces for each bone in the joint are identified using an automated process. **(C)** Joint space volume is identified through a series of morphological operations and subtraction of the original bone volumes. **(D)** Joint space metrics are derived from the 3D map of local joint space widths. The 3D map is shown in pseudo color (left), and as a distribution (right) for the 2nd metacarpal of an RA patient. Reproduced with permission from (13).

HR-pQCT images can also be used to quantify joint space width and volume based on the 3D volume between the bones rather than the 2D analysis that is typically applied to conventional radiographs. Seven publications with joint space analysis were found; all but one has investigated the validity of proposed semi-automatic scripts. Three different algorithms were developed using a similar methodology (11, 13). A consensus method has recently been published by the SPECTRA Collaboration (Figure 10), this method is proposed for universal use in ongoing and future clinical trials of arthritic conditions (70). The established joint space width (JSW) metrics include mean, minimum and maximum JSW as well as standard deviation, asymmetry (maximum/minimum) and joint space volume. A single study has investigated the validity of a JSW script on cadaveric MCP joints at different flexion angles. The study found in order to have reliable joint space measures, the acquisition of the joints had to be < 10 degrees flexion for longitudinal studies (36). Whether the joint space analysis is affected by other factors such as joint swelling, is currently under investigation.

Lastly, two studies evaluated relationships between joint space narrowing assessed from CR and 3D joint space analysis on HR-pQCT. Minimum JSW, JSW standard deviation, and JSW asymmetry assessed by HR-pQCT were associated with SvH joint space score (13, 63).

The minimum joint space width has been shown to be significantly smaller in patients with RA compared to healthy subjects, which is suggestive of a loss of cartilage thickness in the affected patients (13). However, larger studies are needed to reliably investigate group differences.

### Bone Mineral Density and Microstructure

HR-pQCT provides 3D images, so volumetric bone mineral density (vBMD, g/cm^3^) is assessed rather than areal BMD (aBMD) that is reported by dual-energy x-ray absorptiometry (DXA). Microstructural features of trabecular bone, including trabecular thickness, number and separation, have been measured directly and with greater accuracy using the second-generation scanners compared to the first-generation scanner (77). BMD and microstructure imaged by HR-pQCT have been investigated in several different groups of patients and with focus on different joints of interest as well as the typical measurements of the distal radius and distal tibia applied in osteoporosis studies. Most of these studies were cross-sectional, and the majority of studies were done with either RA or PsA patients. One study assessed the relationship of periarticular osteoporosis in the wrist and MCP joint by HR-pQCT imaging with BMD in axial skeletal sites by DXA. Areal BMD in the axial skeleton was moderately correlated with vBMD assessed by HR-pQCT in both the radius and 2nd metacarpal head while microarchitecture only had a weak to moderate correlation in patients with RA (8). Finally, one study investigated the correlation between bone density and bone microstructure parameters measured at the MCP joint and SvH score in patients with RA, but only found a significant correlation between trabecular separation and SvH score (3).

Six cross-sectional studies compared RA patients with healthy subjects. Cortical and trabecular vBMD was significantly lower in female and male patients with RA when compared to healthy subjects at the distal radius, even though no difference in axial aBMD measured by DXA could be observed (17, 24). In the same population, the cortical porosity and trabecular separation were significantly greater, and bone volume fraction significantly lower, in the RA patients (17, 19, 24). Volumetric BMD and microarchitecture were similarly different at the MCP joints in three of four studies. The trabecular vBMD, bone volume fraction and thickness was significantly lower, while trabecular separation was significantly higher in patients with RA compared to healthy subjects (3, 12, 43, 45).

Five cross-sectional studies have investigated bone density and microarchitecture in patients with PsA. At the radius, cortical vBMD was significantly lower, while cortical porosity was significantly higher in PsA patients compared to healthy subjects. By DXA, the PsA patients had significantly higher aBMD at the lumbar spine compared to healthy subjects; this difference, however, became insignificant after adjusting for Body Mass Index (BMI), suggesting BMI may have confounded the relationship (4). Other studies have found a significant loss of periarticular trabecular bone in PsA patients compared to healthy subjects with regards to both vBMD (69) and bone structure (27). Compared to treatment naïve PsA patients, patients with a history of biological disease-modifying anti-rheumatic drugs (DMARD) treatment had significantly higher vBMD, BV/TV as well as greater trabecular number and thickness. No significant differences in bone density or microarchitecture outcomes were observed in patients previously treated with MTX compared to treatment naïve PsA patients (68).

The trabecular number and vBMD were significantly lower, while trabecular separation was significantly higher for PsA patients when compared with RA patients. However, this difference was only seen between ACPA positive RA patients and PsA patients. The ACPA positive RA patients had a significantly longer disease duration. Therefore, this difference is more likely attributed to disease duration rather than the underlying condition (20). In contrast, when comparing patients with various chronic inflammatory diseases (ACPA-positive RA, ACPA-negative RA, Crohn's Disease, ulcerative colitis, psoriasis and PsA) and healthy participants, vBMD differed only between ACPA-positive RA and healthy subjects (56). Together these findings suggest that to date, the bone density and microstructure seems to be affected in different kinds of inflammatory diseases, but the nature of these findings are unclear as the periarticular bone density and microstructure are influenced by other factors independent of the inflammatory nature of the disease, such as genetic factors, sex, physical activity, height, weight, BMI, smoking and alcohol consumption, and daily calcium intake.

### Reproducibility of the Pathological Measures

Reproducibility has been reported for erosion measurements, osteophytes, joint space width, bone mineral density and microstructure (Table 2). Reproducibility is most often presented as Intraclass correlation coefficients (ICC), root mean square coefficient of variance (CV_RMS_%), but also as the coefficient of variation. The reproducibility for bone mineral density, microstructure, and most joint space width metrics are excellent (Table 2). Reproducibility for erosion measures is generally good but does show some variation. Due to the distinct methods used for erosion quantification, these values are hard to compare. Osteophyte assessment has the lowest values for reproducibility (69).

**Table 2:** Reproducibility measures.

**Author, year (ref)**	**Participants**	**Reproducibility feature**	**Method**	**Findings**
**Erosion count**				
Albrecht, 2013 (9)	RA (*n* = 50)	Inter-reader reliability	Erosion count	ICC range: metacarpal head: 0.936–1.00. ICC range: phalangeal Base: 0.948–1.00.
Aschenberg, 2013 (10)	RA (*n* = 40)	Inter-reader reliability	Erosion score	ICC (range): 0.79 (0.57–0.95)
Hecht, 2015 (26)	RA (*n* = 242)	Inter-reader agreement	Erosion count	ICC: [0.87–1.00]
Regensburger, 2015 (28)	RA (*n* = 103)	Inter-reader reliability	Erosion count	ICC [95% CI]: 0.98 [0.98–0.98]
Scharmga, 2016 (34)	RA (*n* = 44); control (*n* = 38)	Intra- and inter-reader reliability	Cortical break count	ICC [95% CI]: 0.61 [0.49–0.70] ICC [95% CI]: 0.55 [0.43–0.65]
Peters, 2017 (40)	RA (*n* = 32); control (*n* = 32)	Inter-reader reliability	Cortical break count 1>0.16 mm, 2>0.33 mm 3>0.53 mm	ICC [95% CI]: 0.91 [0.65–0.97] ICC [95% CI]: 0.81 [0.52–0.92] ICC [95% CI]: 0.52 [0.11–0.78]
Scharmga, 2017 (41)	Cadaver (*n* = 7)	Intra- and inter-reader reliability	Cortical break count	ICC range: 0.52–0.75 ICC range: 0.37–0.55
Peters, 2017 (39)	RA (*n* = 7); control (*n* = 3)	Inter-reader reliability	Cortical break count	ICC [95%CI]: 0.97 [0.90–0.99]
Peters, 2018 (53)	eRA (*n* = 17); undifferentiated arthritis (*n* =4)	Inter-reader reliability and reproducibility	Cortical interruptions count	ICC [95%CI]: 0.96[0.89–0.97] ICC [95%CI]: 0.94[0.89–0.97] ICC [95%CI]: 0.96[0.92–0.98] ICC [95%CI]: 0.94[0.89–0.97]
Scharmga, 2018 (54)	RA (*n* = 39); control (*n* = 38)	Intra- and inter-reader reliability	Cortical interruptions	ICC [95%CI]: 0.69[0.65–0.73] ICC [95%CI]: 0.56[0.49–0.62]
Scharmga, 2018 (55)	RA (*n* = 20); control (*n* = 10)	Intra- and inter-reader reliability	Cortical interruptions	ICC [95%CI]: 0.88 [0.83–0.92] ICC [95%CI]: 0.48 [0.20–0.67]
Simon, 2018 (57)	PsA *(n* = 55); PsO (*n* = 55); control (*n* = 47)	Inter-reader agreement	Erosions number	ICC: 0.96
Berlin, 2019 (60)	Control (*n* = 120)	Inter-reader agreement	Erosion count	ICC [95%CI]: 0.76 [0.62–0.86]
**Erosion score**				
Aschenberg, 2013 (10)	RA (*n* = 40)	Inter-reader reliability	Erosion and osteophyte count and score	ICC (range): 0.79 (0.57–0.95)
**Erosions width and depth**				
Finzel, 2013 (15)	RA (*n* = 20)	Intra- and inter-reader reproducibility	Width and depth	ICC: 0.99 ICC: 0.99 ICC: 0.93 ICC: 0.94
Lee, 2015 (1)	RA (*n* = 16)	Intra- and inter-reader reproducibility	Sum maximum dimension.	ICC: 0.89 ICC: 0.99
Barnabe, 2016 (30)		Inter-reader reliability	Width and depth	CV_RMS_%: 12.3–20.6 CV_RMS_%: 22.2–24.0
Yue, 2017 (46)	RA (*n* = 20); RA (*n* = 20)	Intra- and inter-reader reliability	Width and depth	ICC [95%CI]: 0.99 [0.94–1.00] ICC [95%CI]: 0.99 [0.96–1.00] ICC [95%CI]: 0.98 [0.96–0.99] ICC [95%CI]: 0.98 [0.97–0.99
Keller, 2017 (50)	Control [ACPA+ (*n* = 29); ACPA– (*n* = 29)]	Intrareader reproducibility	Width and depth	CV%: 14.9 CV%:10.8%
Ibrahim-Nasser, 2018 (48)	RA (*n* = 29)	Inter-reader precision	Width and depth	CV_RMS_%: 16.0–20 CV_RMS_%: 17.5–23.4
Yue, 2018 (58)	eRA (*n* = 63); Remission/not remission	Intra- and inter-reader reliability	Width and depth	ICC [95%CI]: 0.97 [0.93–0.99] ICC [95%CI]: 1.00 [0.99–1.00] ICC [95%CI]: 0.98 [0.93–0.99] ICC [95%CI]: 0.92 [0.70– 0.98]
Henchie, 2019 (61)	RA (*n* = 17); PsA (*n* = 17); control (*n* = 12)	Inter-reader precision	Depth.	RMSE: 4+/−3% Precision error: 50 μm
**Erosion volume**				
Töpfer, 2014 (23)	RA (*n* = 18)	Inter-reader precision	Volume (Half-ellipsoid). Volume. (MIAF).	CV_RMS_%: 15.4 CV_RMS_%: 7.78
Regensburger, 2015 (28)	RA (*n* = 103)	Inter-reader reliability	Volume (Half-ellipsoid).	ICC [95% CI]: 0.95 [0.94–0.96]
Töpfer, 2015 (29)	RA (*n* = 22)	Inter-reader precision	Volume. (MIAF).	CV_RMS_%: 6 – 8.3
Figueriredo, 2016 (31)	ACPA+ RA (*n* = 202)	Inter-reader reliability	Volume (Half-ellipsoid).	ICC [95%CI]: 0.96 [0.94–0.97]
Shimizu, 2017 (42)	RA [(*n* = 27) TNFi/MTX *n* = 17/10)	Intra- and inter reader agreement	Volume	CV_RMS_%: 3.43 CV_RMS_%: 3.92
Yue, 2017 (46)	RA (*n* = 20); RA (*n* = 20)	Intra- and inter-reader reliability	Volume	ICC [95%CI]: 0.98 [0.87–1.00] ICC [95%CI]: 0.96 [0.73–1.00]
Keller, 2017 (50)	Control [ACPA+ (*n* = 29); ACPA– (*n* = 29)]	Intrareader reproducibility	Volume (Osirix)	CV%20.6%,
Figueriredo, 2018 (47)	RA (*n* = 65)	Inter-reader reliability	Volume (Half-ellipsoid) Volume (MIAF) Volume (mESE)	ICC [95%CI]: 0.95 [0.92–0.97] ICC [95%CI]: 0.92 [0.79–0.97] ICC [95%CI]: 0.99 [0.99–0.99]
Ibrahim-Nasser, 2018 (48)	RA (*n* = 29)	Inter-reader precision error	Volume (Osirix)	CV_RMS_%: 16.0–20 CV_RMS_%: 17.5–23.4 CV_RMS_%: 14.0–21.2
Peters, 2018 (53)	eRA (*n* = 17); undifferentiated arthritis (*n* =4)	Intra- Inter-reader reliability and Intra- Inter-reader reproducibility	Cortical interruptions (CIDA)	ICC [95%CI]: 0.99[0.99–1.00] ICC [95%CI]: 0.91[0.81–0.96] ICC [95%CI]: 1.00[0.99–1.00] ICC [95%CI]: 0.91[0.81–0.96]
Simon, 2018 (57)	PsA (*n* = 55); PsO (*n* = 55); control (*n* = 47)	Inter-reader agreement	Volume	ICC: 0.90
Yue, 2018 (58)	eRA (*n* = 63); Remission/not remission	Intra- and inter-reader reliability	Volume	ICC [95%CI]: 0.95 [0.79–0.99] ICC [95%CI]: 0.98 [0.83–1.00]
Shimizu, 2019 (65)	RA [(*n* = 28) TNFi+/– *n* = 18/10]	Intra- and Inter-reader reproducibility	Volume (Osirix)	ICC: 0.93 ICC: 0.80
Wu, 2020 (69)	PsA (*n* = 62); control (*n* = 62)	Inter-reader reliability	Volume	ICC [95%CI]: 1.00 [0.99–1.00]
**Erosion surface area**				
Töpfer, 2014 (23)	RA (*n* = 18)	Inter-reader precision	Surface Area (MIAF)	CV_RMS_%: 9.89
Peters, 2017 (40)	RA (*n* = 32); control (*n* = 32)	Inter-reader reliability	Cortical interruptions 1>0.16 mm, 2>0.33 mm 3>0.53 mm	1.ICC [95% CI]:0.93 [0.82–0.97 2.ICC [95% CI]:0.86 [0.67–0.94] 3.ICC [95% CI]:0.21 [−0.15–0.56]
Peters, 2017 (39)	RA (*n* = 7); control (*n* = 3)	Inter-reader reliability	Cortical break Surface area	ICC [95%CI]: 0.98 [0.92–1.00]
Peters, 2018 (53)	eRA (*n* = 17); undifferentiated arthritis (*n* =4)	Intra- Inter-reader reliability and Intra- Inter-reader reproducibility	Cortical interruptions surface area (CIDA)	ICC [95%CI]: 0.95[0.92–0.98] ICC [95%CI]: 0.70[0.41–0.84] ICC [95%CI]: 0.95[0.92–0.98] ICC [95%CI]: 0.70[0.41–0.86]
**Erosion sphericity**				
Töpfer, 2014 (23)	RA (*n* = 18)	Inter-reader precision	Sphericity. (MIAF).	CV_RMS_%: 5.46
**Erosion marginal osteosclerosis**				
Yue, 2017 (46)	RA (*n* = 20); RA (*n* = 20)	Intra- and inter-reader reliability	Marginal osteosclerosis	ICC [95%CI]: 0.98 [0.96–0.99] ICC [95%CI]: 0.97 [0.90–0.99]
Yue, 2018 (58)	eRA (*n* = 63); Remission/not remission	Intra- and inter-reader reliability	Marginal osteosclerosis	ICC [95%CI]: 0.99 [0.91–1.00] ICC [95%CI]: 0.94 [0.73–0.99]
**Osteophyte count**				
Aschenberg, 2013 (10)	RA (*n* = 40)	Inter-reader reliability	Count	ICC (range): 0.79 (0.57–0.95)
Finzel, 2013 (21)	PsA (*n* = 25); HOA (*n* = 25); control (*n* = 20)	Intra- Inter-reader reproducibility	Count	Spearman's rho (0.95–1.00) ICC 0.91
Simon, 2018 (57)	PsA (*n* = 55); PsO (*n* = 55); control (*n* = 47)	Inter-reader agreement	Count	ICC: 0.95
Berlin, 2019 (60)	Control (*n* = 120)	Inter-reader agreement	Count	ICC [95%CI]: 0.96 [0.92–0.98]
**Osteophyte score**				
Aschenberg, 2013 (10)	RA (*n* = 40)	Inter-reader agreement	Score	ICC (range): 0.79 (0.57–0.95)
Finzel, 2013 (21)	PsA (*n* = 25); HOA (*n* = 25); control (*n* = 20)	Intra- and Inter-reader reproducibility	Score	Spearman's rho (0.95–1.00) ICC 0.92
**Osteophyte height**				
Finzel, 2013 (14)	PsA [(*n* = 41) TNFi/MTX *n* = 28/13]; RA (*n* = 43)	Inter-reader reproducibility Intra-reader reliability	Height	*r* = 0.9692 *r* = 0.9722
Finzel, 2013 (21)	PsA (*n* = 25); HOA (*n* = 25); control (*n* = 20)	Inter-reader reproducibility	Height	ICC 0.96
Simon, 2018 (57)	PsA (*n* = 55); PsO (*n* = 55); control (*n* = 47)	Inter-reader agreement	Height	ICC: 0.94
Henchie, 2019 (61)	RA (n = 17); PsA (n = 17); control (n = 12)	Inter-reader precision	Periosteal bone growth height	RMSE: 20+/−13% Precision error:210 μm
Wu, 2020 (69)	PsA (*n* = 62); control (*n* = 62)	Inter-reader reliability	Entesophyte height	ICC [95%CI]: 0.74 [0.42–0.89]
**Osteophyte volume**				
Wu, 2020 (69)	PsA (*n* = 62); control (*n* = 62)	Inter-reader reliability	Entesophyte volume	ICC [95%CI]: 0.99 [0.97–0.99]
**Bone mineral density**				
Fouque-Aubert, 2010 (3)	RA (*n* = 14); control (*n* = 14)	Repositioning and scan/rescan	BMD	CV%: ≤ 1.8
Barnabe, 2013 (12)	RA (*n* = 15); control (*n* = 15)	Intrareader reproducibility	BMD	CV_RMS_%: <0.83
Keller, 2017 (50)	Control [ACPA+ (*n* = 29); ACPA– (*n* = 29)]	Intrareader reproducibility	BMD	CV%: 0.6–3.53
Peters, 2018 (53)	eRA (*n* = 17); undifferentiated arthritis (*n* = 4)	Inter-reader reliability and Intra- Inter-reader reproducibility	BMD	ICC [95%CI]: 0.99[0.99–1.00] ICC [95%CI]: 0.99[0.99–1.00] ICC [95%CI]: 0.99[0.99–1.00] ICC [95%CI]: 1.00[1.00–1.00]
Wu, 2020 (69)	PsA (*n* = 62); control (*n* = 62)	Inter-reader reliability	BMD	CV%: 0.38–1.03
**Bone microstructure**				
Fouque-Aubert, 2010 (3)	RA (*n* = 14); control (*n* = 14)	Repositioning and scan/rescan	Microstructure	CV%: ≤ 12.5
Barnabe, 2013 (12)	RA (*n* = 15); control (*n* = 15)	Intrareader reproducibility	Microstructure	CV_RMS_%: <0.83
Keller, 2017 (50)	Control [ACPA+ (*n* = 29); ACPA– (*n* = 29)]	Intrareader reproducibility	Microstructure	CV%: 0.6–3.53
Peters, 2018 (53)	eRA (*n* = 17); undifferentiated arthritis (*n* =4)	Inter-reader reliability and Intra- and inter-reader reproducibility	Microstructure	ICC [95%CI]: 0.99[0.99–1.00] ICC [95%CI]: 0.99[0.99–1.00] ICC [95%CI]: 1.00[1.00–1.00] ICC [95%CI]: 1.00[1.00–1.00]
Wu, 2020 (69)	PsA (*n* = 62); control (*n* = 62)	Inter-reader reliability	Microstructure	CV%: 0.38–1.03
**Joint space**				
Barnabe, 2013 (12)	RA (*n* = 15); control (*n* = 15)	Intrareader	JSW	CV_RMS_%: 17.1
Burghardt, 2013 (13)	RA (*n* = 16); control (*n* = 7)	Repositioning and scan/rescan	JSV JSW JSW.SD JSW.Min JSW.MAX JSW.AS	CV_RMS_%: 3.5 CV_RMS_%: 2.1 CV_RMS_%: 10.4 CV_RMS_%: 12.5 CV_RMS_%: 2.2 CV_RMS_%: 13.9
Tom, 2016 (36)	Cadaver (*n* = 7)	Repositioning and scan/rescan	JSV JSW JSW.Min JSW.Max	CV_RMS_%: 2.2 CV_RMS_%: 3.8 CV_RMS_%: 8.0 CV_RMS_%: 4.4
Stok, 2020 (70)	RA (*n* = 30)	Repositioning and scan/rescan	JSV JSW JSW.Minimum JSW.Maximum JSW.Asymmetry	ICC [95%CI]: 0.99 [0.98–0.99] ICC [95%CI]: 0.95 [0.91–0.97] ICC [95%CI]: 0.66 [0.36–0.82] ICC [95%CI]: 0.73 [0.50–0.86] ICC [95%CI]: 0.75 [0.54–0.87]

*ACPA, Anti-citrullinated protein antibodies; BMD, Bone mineral density; CV, coefficient of variance; CIDA, Cortical interruption detection algorithm; eRA, Early Rheumatoid arthritis; ICC, Intra-class correlation coefficient; JSW, Joint space width; JSV, Joint space volume; MTX, Methotrexate; MIAF-finger, Medical Image Analysis Framework – Finger; mESE, modified Evaluation Script for Erosions; PsO, Psoriasis; PsA, Psoriatic arthritis; RA, Rheumatoid arthritis; RMSE, root-mean-square error; CV_RMS%_, root mean square coefficient of variance; TNFi, Tumor necrosis factor inhibitors*.

When compared with reproducibility for semi-quantitative scoring systems on MRI (RAMRIS) (78) and CR (SvH) (79), HR-pQCT offers many advantages by using quantitative tools for outcome measurement that require fewer operator inputs. The reliability for erosion count was higher for HR-pQCT than MRI. However, the ICC was excellent for both modalities (9, 28), while only moderate for CR (55). The semi-automated or fully-automated assessments of bone mineral density, microstructure, joints space width and cortical interruptions result in intra-reader and inter-reader differences that are non-existent, or nearly negligible because the analyses are automated, with the exception of an allowance for corrections of bone segmentation. Scan-rescan reproducibility for JSW minimum is similar to that obtained with inter-reader ratings on 2D radiographs (RMSSD 0.18 mm) but far superior for mean JSW (RMSSD 0.05 mm) (80). Intra- and inter-reader reproducibility and reliability for erosion detection and volume are comparable to reproducibility and reliability reported for the erosion domain of the RAMRIS scoring system (9, 28, 81, 82).

### Longitudinal Changes of the Pathological Measures

While the diagnostic uses of HR-pQCT might be limited at present, the research applications are vast. Several clinical trials have been conducted using erosion volume by HR-pQCT as an outcome measure. One of the major advantages is the potential for shorter monitoring times to evaluate erosion progression or repair. Fifteen studies have reported longitudinal changes in the number and/or size of erosions. Of these, three investigated PsA (14, 49, 66), 10 investigated patients with RA (6, 10, 15, 29, 42, 46, 58, 59, 64, 65), and one study examined ACPA+ individuals without arthritis (62). Seven studies investigated intervention with different therapies either as an open-label study or secondary analysis in a randomized controlled trial. Five of the seven studies were done on patients with RA (12, 19, 43, 55, 69), and two in PsA (21, 60). In patients with established RA, two studies of patients with mean disease duration more than 5 years did not detect any change in erosions at 1-year follow-up (10, 64). However, longitudinal changes are diverse. Studies which traced changes of individual erosions in patients receiving care according to treat-to-target strategy over a 1-year period have shown that 15–31% of erosions progress in size, 48–65% were stable in size and 20–21% show partial repair after 1 year (29, 58, 67).

The shortest time interval over which significant changes have been evaluated is 3 months. This study was conducted enrolling patients with RA and a mean disease duration of more than 5 years who were receiving either a TNF inhibitor (TNFi) in combination with methotrexate or methotrexate alone. While no significant changes in MRI outcome measures were observed, a significant increase in erosion volume assessed by HR-pQCT was observed for the methotrexate-treated patients, and those on combination therapy with treatment response had a demonstrated decrease in erosion volume (42). In this same study, joint space did decrease significantly in patients in combination therapy only. Paradoxically, this was not seen in methotrexate-treated patients. However, methotrexate-treated patients had smaller joint space at baseline, which may explained why no change was seen after the 3-month follow-up (42). Change in erosion volume after 3 and 6 months has also been investigated for patients with RA and a mean disease duration of roughly 10 years receiving either alendronate or denosumab together with their regular DMARD treatment (46). No significant change was observed after 3 months. Conversely, erosion volume increased significantly in patients receiving alendronate, while decreasing significantly in patients receiving denosumab after 6 months. While these represent the shortest time interval over which changes in erosion volume and JSW have been observed, two studies have shown decreases in erosion volume in response to tocilizumab in combination with methotrexate for both patients with newly diagnosed RA (59) and established RA (15); this has also been observed for patients with established RA treated with TNFi in combination with methotrexate (6) over a 1-year follow-up. Stabilization in erosion size with therapy has also been demonstrated, in newly diagnosed patients with RA on treatment with a TNFi in combination with methotrexate (59). PsA studies have shown slower erosion changes than RA; no significant progression in erosions was seen in patients with established PsA after 24 weeks (49) or 1-year (14). However, one study found progression of erosion number and size after 5-years in patients with established PsA and a mean disease duration of 14 years (66).

While the measurement of erosion size has been the most commonly used outcome metric, osteophytes and bone quality have also been assessed. Five longitudinal studies investigated changes in osteophytes over time. In RA patients, no significant change in osteophyte number or score was seen after a year (10). However, an 18-month follow-up study did find an increase in both osteophytes number and volume in RA patients in open-label treatment (31). No change in osteophyte size was observed in PsA patients treated with secukinumab for 24 weeks (49). However, in patients treated with MTX and TNFi osteophyte height increased over 1 year (14), and volume increased over 5 years in patients in open-label treatment (66).

Only six longitudinal studies of bone density and microarchitecture in the MCP joint or the radius have been reported. In longitudinal studies of RA or PsA patients without a healthy control group, conducted with 3-month or 1-year follow-up, no significant changes in vBMD or microstructure were observed (42, 49, 58). In one study, greater decreases in vBMD and microstructure over 1 year were observed in RA patients compared to healthy controls (64), while two other studies found comparable decreases in both early RA patients and healthy controls (37, 62). Differences in disease duration, treatment status, and the anatomic site measured may explain the variable findings across studies. More extensive longitudinal studies are needed to reveal the critical anatomic locations, time course, and mechanisms of bone loss that should be strategically targeted for observation.

Combined with advances in image processing and image registration, individual spatially localized changes can be monitored with high sensitivity and reliability (Table 2) (64). To date, the published studies report data for the joint region captured in the field of view, and not for the local changes such as the peri-articular region. Furthermore, these techniques can be applied where only subtle changes are anticipated, such as understanding bony damage progression in the presence of subclinical inflammation.

### Early Detection of Articular Damage in Inflammatory Arthritis by HR-pQCT Imaging

The sensitive detection of erosive damage and bony proliferation suggests that HR-pQCT may have utility in the early detection of articular damage in inflammatory arthritis. For conventional radiographs, erosions have been observed to be prevalent in ~50% of RA patients at clinical diagnosis, and up to 60% of patients have erosions after 1 year (83). Therefore, the higher sensitivity of HR-pQCT compared to conventional radiographs is promising not only in assessing longitudinal changes but may also be useful for early detection in patients where the diagnosis is suspected but cannot be made clinically. However, the ability to image multiple joints is limited by the acquisition speed. Although many erosions occur in the standard scan sites of the 2nd and 3rd metacarpals of the dominant hand, other joints will be missed. Further, the contrast is insufficient to detect inflammation (synovitis and osteitis, also referred to as bone marrow oedema in RA patients) believed to be key precursors to clinical disease. Nonetheless, two cross-sectional studies found a significantly thinner trabecular bone in the MCP joints of ACPA positive individuals with arthralgia compared with ACPA negative healthy individuals (18, 50). Kleyer et al. also observed significantly lower trabecular number, bone volume fraction and vBMD in ACPA positive individuals with arthralgia compared with ACPA negative healthy individuals (18), suggesting that periarticular bone loss may be an early sign of clinical disease. Changes to bone mineral density and microstructure, however, is very non-specific to arthritis, as possible changes can be attributed to many other factors. Therefore, the use of bone mineral density and microstructure for early detection of articular damage in inflammatory arthritis is still limited. The number and volume of erosions have been shown to increase over a 1-year period in ACPA positive individuals with arthralgia but with no sign of arthritis and when compared with an ACPA negative control group (62). Therefore, erosion number and size may be a sensitive measure for early RA progression.

## Current Limitations and Future Directions for Improvement

### Availability of an Accessible Tool for Erosion Quantification

Clinical studies have been successfully performed using HR-pQCT and a variety of custom software packages for analysis. The detection of clinical erosive features is demonstrated, as well as the reproducibility and sensitivity to measure change. However, while erosion volume quantification shows clear promise as an outcome measure, one of the challenges has been the heterogeneity of approaches to identify erosions and measure their size. This also includes distinguishing erosions from other physiological features. An ideal tool would require minimal operator input but allow corrections when necessary. Furthermore, current approaches have been built with a variety of proprietary and/or custom software packages which are typically only accessible to individual research groups. To improve reproducibility and consistency of definitions, as well as the widespread adoption of semi-automated techniques, a new approach must be developed in an open-source, user-friendly software platform. Machine learning approaches may also better automate erosion detection (84).

### Segmentation to Assess the Cortical and Trabecular Bone Density and Microstructure

The current standard approach for segmentation to assess bone mineral density and microstructure is a semi-automated slice-by-slice hand contouring approach or semi-automated edge detection algorithm (85). The algorithms were developed for the distal radius and tibia with the assumption that cortical bone is thicker and denser than trabecular bone, which is not necessarily appropriate for the MCP and PIP joints (86). As well, joints with low bone mineral density and high cortical porosity, such as seen in advanced RA, have proven problematic for the algorithms. The presence of cortical breaks and erosions is also problematic for the existing segmentation approaches, i.e., there exists no consensus as to whether the site of an erosion should be included or excluded. Another important consideration for the examined joint is how much of the bone should be segmented, some have used a volume of interest that spans the majority of the metaphysis, and is standardized to be 10% of the metacarpal length (45). However, presently, there is no consensus on this matter.

To date, significant differences in bone mineral density and microstructure between patients with inflammatory arthritis and healthy controls were primarily reported for the trabecular bone compartment. It is possible that further development of the segmentation algorithms and the imaging resolution could improve segmentation accuracy and might reveal greater differences in the cortical bone of inflammatory arthritis patients.

### Motion Compensation

One disadvantage of HR-pQCT imaging is that the lengthy, but highly sensitive acquisition process renders it susceptible to motion artifacts, which can impair the ability to accurately and precisely quantify bone and joint outcomes (87, 88). To date, the number of scans excluded for patient motion is rarely reported with sufficient detail, and as such, it is challenging to know the impact of motion on outcomes.

Motion artifact is particularly problematic for longitudinal studies or when implementing more advanced analyses, such as quantifying bone structure, formation, and resorption (89, 90). While this technique holds promise to identify erosions undergoing repair or progression (64), the technique is currently limited to cross-sectional studies because motion artifact typically occurs in the multi-acquisition scan (multi-stack) for joints, and might falsely detect repair or progression. A partial solution to this problem has been implemented by overlapping the acquisitions. However, this simply serves to smooth the effects of motion on scan quality rather than eliminate it (90). Ideally, existing motion compensation algorithms could be implemented in HR-pQCT image reconstruction software to reduce data loss and maximize the utility of acquired data (91, 92).

### Joints of Interest

Conventional radiographs assess erosions in 16 joints in each hand and six joints in each foot. Presently, the image acquisition protocol recommended by the SPECTRA Collaboration for the MCP joints specifies that at a minimum, both the 2nd and 3rd MCP should be imaged (93). There is currently no standard image acquisition protocol for the PIP joints. In spite of this, a limited number of published studies have investigated the PIP joint in a similar fashion as the MCP joints (12, 34, 49, 52, 54, 55, 60, 64). The wrist is also examined in multiple studies with the intention of evaluation of bone mineral density and microstructure of the radius, and JSW in the wrist (13). Still, no protocol for assessing erosions in the radius have been published. Whether there is any benefit to include other joints such as the knee, elbow, ankle or foot remains unclear, but are accessible for HR-pQCT imaging. For the HR-pQCT to be a viable modality for assessing progression in clinical practice, further research to explicitly investigate the number and which specific joints needed for the HR-pQCT scanner to have an added benefit compared to CR are needed.

### Disadvantages of HR-pQCT Scanning and the Limited Field of View

The HR-pQCT scanner is not widely available to most researchers or clinicians. Presently, HR-pQCT scanners are only developed by Scanco Medical (Bruttisellen, Switzerland). Worldwide only 93 HR-pQCT scanners are installed (46 of the first-generation and 47 of the second-generation), and about 20 research groups use the technology in rheumatological research, this limits the feasibility of implementing the method in clinical practice at this time. However, the existing HR-pQCT models use cone-beam configurations (conical x-ray beam which projects onto a flat panel detector), the term “cone-beam CT” has been adopted in reference to scanners with a very large detector that commonly uses this hardware configuration. Cone-beam CT provides higher spatial resolution at lower costs than conventional multi-detector CT systems (94). Acquisition times can be much faster (<1 min vs. 6 to 9 min) than HR-pQCT due to the larger detector sizes. Improvements to image reconstruction can advance the accuracy of bone microarchitectural measurement to be comparable with HR-pQCT (95, 96). Therefore, cone-beam CT could be a viable modality to complement HR-pQCT imaging, thereby increasing access. The outcome measures described throughout this review could be widely implemented for cone-beam CT. Another limitation of exclusively using HR-pQCT for inflammatory arthritis is the inability to visualize soft tissue and inflammation. Dual-energy CT scans acquire projections at two different energy levels to take advantage of differing attenuation coefficients for different tissues at each energy (97–99). Approaches have been implemented in commercial multi-detector CT scanners (100) and have been used to identify tophi and other crystal arthropathies (101–103), as well as more recently to identify bone marrow edema (104, 105). While not currently available in HR-pQCT scanners, there is the potential that future extremity CT scanners will additionally be able to quantify soft tissue and inflammatory abnormalities.

### Using Multi-Modal Imaging to Link Erosive Damage to Inflammatory Markers

Investigators have proceeded with evaluating if HR-pQCT images and findings can be used in conjunction with those obtained using US or MRI to provide a comprehensive overview of soft tissue and bone in the same joint. A complimentary paper on this topic is included in this issue (Tse et al., submitted). In particular, the presence of hyperintense signals on MRI linked to inflammation in RA patients, such as osteitis (106) and synovitis is associated with more pronounced erosive damage in patients with RA (52, 54). In the radius of patients with RA, bone mineral density, trabecular thickness and number were significantly higher, while trabecular separation was lower in regions with MRI-defined osteitis than without (22). Similarly, US Doppler-positive RA patients identified with ultrasound had a lower trabecular number and vBMD alongside higher trabecular separation and distribution of trabecular separation in the MCP joints compared to Doppler negative patients (51).

### Education for Patients and Students

Lastly, the use of HR-pQCT imaging as possible pedagogical tool should not be overlooked. The spatial and high-resolution imaging are useful for developing 3D models of the periarticular changes of the joint. 3D printed prototypes of arthritic and healthy joints from HR-pQCT data have been investigated as a tool to demonstrate joint disease to address an eventual gap in patient education and disease understanding (38). The HR-pQCT data have also been used with Virtual Reality application for student training (107).

## Conclusions

In conclusion, owing to the ability to detect change over time periods as short as 3 months, there is a great capacity to utilize HR-pQCT measurements as outcomes in future clinical trials. In addition, HR-pQCT provides uniquely high *in-vivo* sensitivity for erosion detection compared to other modalities. The erosion measures, joint space narrowing, bone density and microstructure have high to excellent reproducibility, whereas osteophytes measure only showed moderate to high reproducibility. The highly sensitive assessment of bone microstructure and damage has the potential to better understand bone changes, particularly in the early stages of inflammatory diseases. Finally, new research developments will improve the accessibility of scanners, the field of view of the imaging, analysis tools such as automatic methods for longitudinally evaluating distinct erosions, as well as the versatility for measuring tissues other than only bone.

## Data Availability Statement

The original contributions presented in the study are included in the article/Supplementary Material, further inquiries can be directed to the corresponding author/s.

## Author Contributions

RK-J performed the systematic literature review. RK-J and SM drafted the manuscript. All authors contributed conception and design of the review and contributed to manuscript revision, and read and approved the submitted version.

## Conflict of Interest

The authors declare that the research was conducted in the absence of any commercial or financial relationships that could be construed as a potential conflict of interest. The reviewer AK declared a past collaboration with one of the authors SF to the handling editor.
