# Supplementary material for: High-Resolution Peripheral Quantitative Computed Tomography for Bone Evaluation in Inflammatory Rheumatic Disease
Source: Front Med (Lausanne). 2020 Jul 15;7:337. doi: 10.3389/fmed.2020.00337 (PMC7381125; doi:10.3389/fmed.2020.00337)
Supplement: Supplementary file 1 [file Data_Sheet_1.PDF]

APPENDIX 1

Pubmed - 250

("Metacarpophalangeal Joint"[Mesh] OR "Wrist"[Mesh] OR "Wrist Joint"[Mesh] OR metacarpophalangeal Joint[Title/Abstract] OR MCP*[Title/Abstract] OR Wrist*[Title/Abstract] OR wrist Joint*[Title/Abstract] OR "Radius"[Mesh] OR Radius*[Title/Abstract]) AND ("Tomography, X-Ray Computed"[Mesh] OR HR-pQCT*[Title/Abstract] OR HRpQCT*[Title/Abstract] OR HR pQCT*[Title/Abstract] OR pQCT*[Title/Abstract] OR high-resolution peripheral quantitative computed tomography*[Title/Abstract] OR high resolution peripheral quantitative computed tomography*[Title/Abstract] OR Xtremect*[Title/Abstract] OR xtreme ct*[Title/Abstract] OR microct*[Title/Abstract] OR micro ct*[Title/Abstract] OR micro-ct*[Title/Abstract] OR high-resolution computed tomography*[Title/Abstract] OR high resolution computed tomography*[Title/Abstract] OR high resolution computed tomography*[Title/Abstract]) AND ("Arthritis"[Mesh] OR Arthritis*[Title/Abstract]) AND ("English"[Language])*

Embase - 80

(('metacarpophalangeal joint':ti,ab,kw OR wrist:ti,ab,kw OR radius:ti,ab,kw) AND 'hr pqct':ti,ab,kw OR hrpqct:ti,ab,kw OR 'hr pqct':ti,ab,kw OR 'high resolution peripheral quantitative computed tomography':ti,ab,kw OR 'high-resolution peripheral quantitative computed tomography':ti,ab,kw OR 'micro-ct scanner':ti,ab,kw OR 'x-ray computed tomography':ti,ab,kw) AND arthritis:ti,ab,kw AND english:la AND [1966-2020]/py
